# Age-Friendly Community Education: Fostering Health Behavior Change in a Medically Underserved Community

**DOI:** 10.1093/geroni/igaa057.1776

**Published:** 2020-12-16

**Authors:** Katherine Thompson

**Affiliations:** University of Chicago Medicine, Chicago, Illinois, United States

## Abstract

The University of Chicago GWEP, SHARE Network (Supporting Healthy Aging Resources and Education), is located on the South Side of Chicago, an urban medically underserved community. SHARE Network partners with a variety of community based organizations (CBOs) to support Age Friendly care through an Age Friendly Community Education Curriculum. In a one year period, 28 events were held in 13 CBO sites. Each of these events addressed the 4Ms of Age Friendly care with a specific content focus on Mentation (5 events), Mobility (5), Medications (4), and What Matters (10), and 4 events focused on multiple domains. There were 458 attendees, 290 completed surveys (63% response rate). Eighty-eight percent of participants planned to make a healthy behavior change after participation. Age Friendly Community Education can complement health system transformation to lead to health behavior change for community-dwelling older adults in medically underserved communities.

